# Assessment of the Concentration of Endogenous Factors Regulating Angiogenesis, VASH-1 and VEGF-A, in the Blood Serum of Patients with Neuroendocrine Neoplasms

**DOI:** 10.1155/2022/9084393

**Published:** 2022-03-10

**Authors:** Janusz Strzelczyk, Monika Wójcik-Giertuga, Piotr Cuber, Krzysztof Biernacki, Beata Kos-Kudła, Joanna Katarzyna Strzelczyk

**Affiliations:** ^1^Department of Endocrinology and Neuroendocrine Tumors, Department of Pathophysiology and Endocrinology, Faculty of Medical Sciences in Zabrze, Medical University of Silesia in Katowice, 35 Ceglana St., 40-514 Katowice, Poland; ^2^Natural History Museum, Core Research Laboratories, Molecular Biology, Cromwell Road, London SW7 5BD, UK; ^3^Department of Medical and Molecular Biology, Faculty of Medical Sciences in Zabrze, Medical University of Silesia, 19 Jordana St., 41-808 Zabrze, Poland

## Abstract

Neuroendocrine neoplasms (NENs) constitute about 2% of all malignant neoplasms, and the angiogenesis process in these tumors is still of a great interest. Vasohibin-1 (VASH-1) is an angiogenesis inhibitor, while vascular endothelial growth factor A (VEGF-A) is one of the main factors promoting vascular formation. The subject of this study was to assess serum concentration of these factors in patients with diagnosed NEN and in control group. *Methods*. The study group consisted of 120 patients with diagnosed NENs, while the control group consisted of 69 healthy volunteers. The concentrations of VASH-1 and VEGF-A in serum were tested using the ELISA. We also analyzed the association of the concentration of these factors with demographic data (e.g., age and gender), body mass index (BMI), primary tumor location, histological grade, metastasis, clinical staging, selected biochemical parameters and markers of NENs, and information on smoking habits. *Results*. The mean concentration of VASH-1 was 218.8 ± 359.8 pg/ml in the study group and 973.1 ± 1239.4 pg/ml in the control group, that difference was statistically significant (*p* < 0.05). In the NEN group, the highest concentration of VASH-1 was in patients with pancreatic NENs in relation to NENs with different location of the primary tumor (*p* < 0.05). Negative correlation was found between the concentration of VASH-1 and serotonin (*r*_*S*_ = −0.19, *p* < 0.05). No statistically significant differences were observed for VEGF-A (*p* = 0.658). *Conclusions*. Patients with NENs showed lower serum level of VASH-1 in comparison to healthy volunteers. The highest level of VASH-1 was observed in tumors localized in pancreas. This might reflect the relevant function of VASH-1 in NENs and requires further evaluation to further knowledge of angiogenesis in NENs. Furthermore, the serum concentration of VEGF-A showed no statistical differences and probably does not have diagnostic value in this group of patients.

## 1. Introduction

Neuroendocrine neoplasms/tumors (NENs/NETs) constitute about 2% of all malignant tumors, and the angiogenesis process in these tumors is still of a great interest. They originate from cells of the diffuse endocrine system (DES). The detection level of neuroendocrine neoplasms has been increasing in recent years [1, 2]. About 70% of them are located in the gastrointestinal tract (gastroentero-pancreatic neuroendocrine neoplasm (GEP-NEN)). The classification of gastrointestinal neuroendocrine neoplasms is based on the assessment of their histological maturity. According to the recommendations of the American Joint Committee on Cancer/Union for International Cancer Control (AJCC/UICC) and the WHO classification from 2017, a group of highly differentiated neoplasms was distinguished. The following categories were created based on Ki-67 proliferation index: NET G1 (with the Ki-67 less than 3%), NET G2 (with the Ki-67 proliferation index ranging from 3% to 20%), and NET G3 (with Ki-67 between 20% and 55%); and a group of low-differentiated cancers: NEC (with a Ki-67 proliferation index above 55%). This group also includes large-cell and small-cell carcinomas [2–5]. The respiratory system is the second most common primary localization site for NENs, which constitute approximately 20% of all lung neoplasms [6]. According to the 2015 WHO classification of lung and pleural neoplasms, neuroendocrine neoplasms of the respiratory system (broncho-pulmonary neuroendocrine tumor (BP-NET)) can be divided into 4 groups: typical (TC) and atypical (AC) carcinoids, large cell neuroendocrine carcinoma (LC-NEC), and small cell lung cancer (SCLC) [7]. SCLC is the most common BP-NET [6], but these patients were not included in our study.

Angiogenesis is a complex process of blood vessel formation, which is regulated by stimulating factors, such as VEGF-A (vascular endothelial growth factor A) and inhibitory factors, such as VASH-1 (vasohibin-1). When a tumor reaches a size of approximately 1-2 mm, it needs the necessary nutrients to continue to develop [8]. VASH-1, which is an angiogenesis inhibitor [9, 10], is encoded by the *VASH-1* gene consisting of 7 exons located on the 14q24.3 chromosome [11]. *VASH-1* is stimulated by the VEGF, which is a homodimeric glycoprotein with a molecular weight of approximately 45 kDa. It is a key mediator of angiogenesis [12]. This adequate blood supply to the tumor enables its growth and initiation of metastasis. Using its own or various other mechanisms for this purpose, it stimulates the development of its own blood vessels through the synthesis of angiogenic factors. The tumor blood vessels are disordered and have abnormal shape and size with increased wall permeability [8]. Neoplastic angiogenesis has always raised a great interest, as it can serve as a potential target for treatment.

The influence of angiogenic factors on angiogenesis and the neoplastic process has been extensively studied in malignant neoplasms of various locations and less in patients with neuroendocrine neoplasms. The aim of the study was the quantitative determination of the VASH-1 and VEGF-A concentrations in serum of patients with neuroendocrine tumors and to evaluate its association with the demographic data (age, gender), BMI, the location of the primary tumor, histological grade, and metastasis, as well as selected biochemical parameters (glucose, total cholesterol, triglycerides) and risk factor (smoking). The concentrations of selected specific and nonspecific markers of neuroendocrine neoplasms, such as chromogranin A, serotonin, and 5-hydroxyindoleacetic acid, were also assessed in patients with NEN.

## 2. Materials and Methods

### 2.1. Patients

The study group consisted of 120 patients with neuroendocrine tumors, while the control group was comprised of 69 healthy volunteers. The mean age of the patients in the study group was 57.7 ± 14.0 and in the control group 45.3 ± 15.2. The main inclusion criterion for the study group was a diagnosis of neuroendocrine tumors. Signed consent to participate in the study and confirmed medical family history were also the criteria for inclusion in the study. Most of the patients in the study group were before the initiation of treatment; only 13 patients in this group were treated with a somatostatin analogue. In the case of our study, patients declared that they did not consume alcohol more than occasionally. Exclusion criteria for both groups were pregnancy, lactation, unsure medical family history, age less than 18, and lack of informed consent to participate in the study. None of the individuals from the control group had a history of any cancer disease.

This study was conducted in accordance with the Declaration of Helsinki and Good Clinical Practice guidelines. The Ethics Committee of the Medical University of Silesia (no. KNW/Nr PCN/0022/KB1/98/I/19/20) approved this study. All patients were recruited at the Department of Endocrinology and Neuroendocrine Tumors, Medical University of Silesia. 100 patients with GEP-NET and 20 patients with BP-NET were distinguished in the study group. The characteristics of the groups are presented in Table 1.

The serum concentrations of selected angiogenic factors, such as VASH-1, and the concentration of VEGF-A in the study and in the control groups were assessed using the ELISA method described below. The peripheral blood samples were taken from all patients from the study and control groups who consented to participate in the study. After clotting, the samples of the blood were centrifuged for 15 min at 1000 × g, and serum was collected. Serum was stored at -80°C for further analysis. Information on the levels of chromogranin A, 5-hydroxyindole acetic acid, serotonin, glucose, total cholesterol, and triglycerides levels, as well as information on age, BMI, gender, smoking, tumor localization, histological grade, metastasis, and clinical staging of the patients was accessed through patients' hospital records.

### 2.2. Enzyme-Linked Immunosorbent Assay (ELISA)

ELISA was performed with commercially available ELISA kits: Human VASH-1 ELISA kit (cat. No: CSB-EL025794HU, Cusabio, USA) and Human VEGF-A ELISA kit (cat. No: 650.080.192, Diaclone, France) according to the procedures recommended by the manufacturers. In case of VEGF-A test, all serum samples were diluted 1 : 2 with sample diluent. For both ELISA tests VASH-1 and VEGF-A, plates were read by Bio-Tek *μ*Quant Universal Microplate Spectrophotometer (Bio-Tek, USA), using 450 nm as the primary wavelength and the KCJunior (Bio-Tek, USA) data analysis software. The absorbance was transformed to concentration (pg/ml). All standards and serum samples were run in duplicates. All immunoassays were performed at the Department of Medical and Molecular Biology, Faculty of Medical Sciences in Zabrze, Medical University of Silesia in Katowice.

### 2.3. Statistical Analysis

The comparison of concentrations of VASH-1, VEGF-A, age, BMI, gender, smoking, and glucose between the study and the control groups was performed using Kruskal-Wallis test. Comparison of concentrations of VASH-1, VEGF-A, age, gender, BMI chromogranin A, serotonin, 5-hydroxyindoleacetic acid, glucose, total cholesterol, triglycerides, in subgroups based on smoking, tumor localization, clinical stage, histological grade, metastasis, and proliferation index (Ki-67) was performed using Kruskal-Wallis test. Correlation coefficient between concentration of VASH-1, VEGF-A, age, BMI, chromogranin A, serotonin, 5-hydroxyindoleacetic acid, glucose, total cholesterol, triglycerides, clinical stage, histological grade, and proliferation index (Ki-67) was calculated using Spearman rank correlation coefficient (*r*_*S*_). In all tests, the significance threshold was set at *α* < 0.05. Results for groups and subgroups were presented as mean value and standard deviation. Statistical analysis was performed using the Microsoft Excel (Microsoft) and Statistica v. 13.36.0 (StatSoft, Kraków, Poland) software.

## 3. Results

The study involved 120 patients diagnosed with GEP-NET and BP-NET neuroendocrine neoplasms and 69 people in the control group. The location of the primary lesion in GEP-NETs included the pancreas, duodenum, small intestine, rectum, appendix, stomach, and unknown primary site. Considering the histological grade, in the case of patients diagnosed with GEP-NET, we distinguished NET G1 (67 patients), NET G2 (29 patients), and NET G3 (4 patients). In case of BP-NET patients, the following histological types were present: typical carcinoid (14 patients), atypical carcinoid (5 patients), and large cell neuroendocrine carcinoma (1 patient). According to the European Neuroendocrine Tumour Society (ENETS) and the American Joint Committee on Cancer (AJCC) TNM staging systems, 46 patients in our study were in stage I, 16 patients in stage II, 22 patients in stage III, and 35 patients in stage IV [2]. Correlations were found in the study group between age and glucose concentration (*r*_*S*_ = 0.22, *p* < 0.05) and between BMI and age (*r*_*S*_ = 0.33, *p* < 0.05), as well as between BMI and glucose and triglyceride levels (*r*_*S*_ = 0.23 and *p* < 0.05 and *r*_*S*_ = 0.39 and *p* < 0.05, respectively). More correlations were also demonstrated between cholesterol and triglycerides (*r*_*S*_ = 0.33, *p* < 0.05) and between triglycerides and glucose (*r*_*S*_ = 0.24, *p* < 0.05). Additionally, clinical stage showed correlations between BMI (*r*_*S*_ = −0.21, *p* = 0.05), chromogranin A (*r*_*S*_ = 0.22, *p* = 0.05), serotonin level (*r*_*S*_ = 0.31, *p* = 0.05), 5-hydroxyindole acetic acid level (*r*_*S*_ = 0.31, *p* < 0.05), histological grade (*r*_*S*_ = 0.31, *p* = 0.05), and proliferation index (Ki-67) (*r*_*S*_ = 0.37, *p* < 0.05). There was also a correlation between the concentration of chromogranin A and the concentrations of other markers of neuroendocrine neoplasms, such as serotonin and 5-hydroxyindole acetic acid (*r*_*S*_ = 0.38 and *p* < 0.05 and *r*_*S*_ = 0.33 and *p* < 0.05, respectively) and between serotonin and 5-hydroxyindole acetic acid (*r*_*S*_ = 0.4, *p* < 0.05). Further correlations were found between the proliferation index (Ki-67) and the concentrations of chromogranin A and serotonin (*r*_*S*_ = 0.22 and *p* < 0.05 and *r*_*S*_ = 0.18 and *p* < 0.05, respectively). The results are presented in Table S1. These associations were previously described in the literature and will not be discussed here.

### 3.1. VASH-1

The mean concentration of VASH-1 was 218.8 ± 359.8 pg/ml in the study group and 973.1 ± 1239.4 pg/ml in the control group, and the difference was statistically significant (*p* < 0.05) (Table 2). There were no statistically significant differences in the concentration of VASH-1 in relation to gender, age, and BMI.

The highest concentration of VASH-1 was found in patients with pancreatic NETs as compared to neuroendocrine neoplasms with a different primary localization site (*p* < 0.05). There were no statistically significant differences in the concentration of VASH-1 depending on the histological grade and the metastasis, including metastasis to the lymph nodes, liver and bone, and clinical stage. Also, no statistically significant differences were found between the selected biochemical parameters (total cholesterol and triglycerides) or smoking habits and the concentration of VASH-1 in the study group. A weak negative correlation was found in the study group between the concentration of VASH-1 and the concentration of serotonin (*r*_*S*_ = −0.19, *p* < 0.05). However, no correlation was found between VASH-1 concentrations and other markers of neuroendocrine neoplasms, such as chromogranin A and 5-hydroxyindole acetic acid. Moreover, a weak correlation was found between the concentration of VASH-1 and the concentration of glucose (*r*_*S*_ = 0.19, *p* < 0.05).

### 3.2. VEGF-A

The mean concentration of VEGF-A was 326.3 ± 309.4 pg/ml in the study group and 290.7 ± 175.5 pg/ml in the control group. There were neither statistically significant differences between the concentrations of VEGF-A in the study and control groups (Table 2) nor there were statistically significant differences in the concentration of VEGF-A in relation to gender, age, BMI, and the location of the primary tumor.

There were no statistically significant differences between the concentration of VEGF-A and the histological grade, and metastasis, including metastasis to the lymph nodes, liver, and bone, and clinical stage. No statistically significant differences between the selected biochemical parameters (such as glucose, total cholesterol, and triglycerides) as well as the markers of neuroendocrine neoplasms (chromogranin A, serotonin, and 5-hydroxyindole acetic acid) and the concentration of VEGF-A were found. There was also no statistically significant difference between VEGF-A concentration and cigarette smoking observed. The results are presented in Table S1.

### 3.3. VASH-1 and VEGF-A

There was no correlation between VASH-1 and VEGF-A in the study group (*r*_*S*_ = −0.08, *p* = 0.359). However, there was a negative correlation between VASH-1 and VEGF-A (*r*_*S*_ = −0.49, *p* < 0.05) in the control group. For both groups, no statistically significant differences were found between VASH-1 and VEGF-A and gender, age, and BMI. The results are presented in Table S1 and Table S2.

As shown by statistical analyses in the study group, the treatment with somatostatin analogue did not have any effect on the concentration of VASH-1 and VEGF-A. The treatment showed a significant effect only on the concentration of 5-hydroxyindole acetic acid (*p* < 0.05), without any effect on other parameters. However, these analyses are not reliable due to the small number of patients using somatostatin analogues compared to those who do not.

## 4. Discussion

Neoplastic angiogenesis is still not fully understood process, and it still attracts a lot of attention. Endothelial cells (EC) are the lining of blood vessels, and their role is not only to transport but also to secrete many biologically active substances. VASH-1 is an angiogenesis inhibitor produced by endothelial cells. Apart from VASH-1, vasohibin-2 (VASH-2) produced by cells other than endothelial is distinguished as a homologue of VASH-1, showing the opposite function promoting angiogenesis in animal models [11]. This was confirmed in a study by Hosaka et al., where VASH-1 was restricted to endothelial cells and its expression correlated with the expression of VEGF, FGF-2 (basic fibroblast growth factor 2), and HIF-1*α* (hypoxia-inducible factor 1 alpha) in tumor cells. These studies carried out on mice showed that endogenous VASH-1 is ineffective in inhibiting the “sprouting” of new tumor blood vessels, and only exogenous administration of VASH-1 broke this barrier and inhibited the angiogenesis process [9]. Kosaka et al. demonstrated that VASH-1 may be used as a prognostic factor for disease progression in prostate cancer [13]. In the case of patients with prostate cancer, it has been shown that the expression of VASH-1 reflected the degree of malignancy within this gland [14]. A study by Ninomiya et al. presents that high expression of vasohibins (VASH-1 and VASH-2) in tumor vessels in patients with esophageal cancer is an independent marker of poor prognosis [15]. Also in ovarian cancer high, VASH-1 expression correlates with other angiogenic factors and Ki-67 expression and may also be a prognostic factor [16]. In colorectal cancer, VASH-1 shows a positive correlation with the clinical advancement of the tumor, while in this case no correlation with tumor differentiation was found. Patients with high VASH-1 expression presented significantly worse overall survival (OS) and progression-free survival (PFS) rates than patients with low VASH-1 expression. Similarly, another study showed that high VASH-1 expression in colorectal cancer increased the malignancy potential and promoted the formation of metastasis [17, 18]. VASH-1 has also been shown to be of importance in the case of lung cancer [19, 20].

To our knowledge, there is no published data on the assessment of serum VASH-1 in patients with neuroendocrine neoplasms. Our study showed a significantly higher concentration of VASH-1 in the blood serum of the pancreatic NETs as compared to the other tumor locations. Only few reports so far concern the assessment of VASH-1 in tumor tissue itself. Yazdani et al. examined the structure of blood vessels in the tumor by immunohistochemical staining in 135 cases of gastrointestinal neuroendocrine neoplasms. VASH-1/CD31 has been shown to be an excellent immunohistochemical marker for the characterization of neovascularization in neuroendocrine neoplasms. VASH-1/nestin expression score, representing the rate of new vessel proliferation, was significantly higher in pancreatic NETs than in nonneoplastic islets. Moreover, it has been shown that, according to the WHO classification (from G1 to G2), its expression increases, but it is decreased in neuroendocrine cancers, although the differences did not reach statistical significance [21]. The association between the location of the tumor in the pancreas and the concentration of VASH-1 and VASH-2 was also found in other malignant neoplasms of the pancreas. In the case of pancreatic adenocarcinoma, based on the analysis of tissue models in mice, high VASH-2 concentration has been shown to be associated with the metastatic process and a worse prognosis [22]. Other authors have also shown that in pancreatic cancer, VASH-1 expression is regulated by TGF-*β*/BMP (transforming growth factor-beta/bone morphogenetic protein) signaling. The abovementioned mechanisms might play a role in antiangiogenic therapy in pancreatic cancer [23].

Our study showed a weak correlation between glucose concentration and VASH-1. The literature to date describes that in patients with type 2 diabetes, both VASH-1 and UACR (Urine Albumin-to-Creatinine Ratio) are positively correlated with the concentration of glycated hemoglobin (HbA1C) [24]. According to other authors, no such relationship was demonstrated, and fasting glycemia and HbA1c did not correlate with the concentration of VASH-1 in blood plasma and urine [25]. The study also found a weak negative correlation between the concentration of serotonin, one of the selected markers of neuroendocrine neoplasms, and the concentration of VASH-1. It is interesting that in the case of pancreatic NETs, the so-called “neuroendocrine tumors paradox” has been described, which was based on a higher expression of selected angiogenic factors, such as VEGF, in benign pancreatic neuroendocrine tumors, and was associated with a better prognosis [26]. This seems to be important in the case of the VASH-1 and serotonin correlation, as usually low values of neuroendocrine tumor markers in clinical practice are associated with a better prognosis in these patients. However, in the case of gastrointestinal neuroendocrine neoplasms, the role of VEGF is still not fully understood [26].

Correlations found by our study among other NEN markers, such as serotonin (5HT), 5-hydroxyindole acetic acid (5HIAA), chromogranin A (CgA/ChgA), and proliferation index (Ki-67), were statistically significant, which confirms their previously proven role in cancerogenesis and usefulness in diagnostics. These markers were investigated and proven before to be strongly correlated with tumor burden and were also proven to often mirror the behavior of one another [27–31]. On the other hand, there are publications reporting lack of any association among them, and the diagnostic utility of some of them, e.g., CgA, is being questioned [32, 33]. Correlation especially between serotonin and 5HIAA is understandable as the latter is a metabolite of the former [27, 31]. Chromogranin A is a protein produced and released by tumor cells and a good marker generally associated with all neuroendocrine tumors [29, 30]. It is associated with other hormones suggesting it may be involved in their regulation [28], and it may also be associated with cell adhesion in metastatic stages of the disease [29, 30]. Still, its function and involvement in cancerogenic processes are not fully understood [34]. The value of the Ki-67 is undeniable; however its correlation with the abovementioned markers is not very clear. Some authors found no such correlation [32], while others suggest its existence [35]. We found such correlation in our study, which indicates the need for further meta-analysis to investigate this issue.

VASH-1 is stimulated by factors such as VEGF and FGF-2, one of the most important angiogenic factors. VASH-1 expression was reported to be positively correlated with VEGF-A in colorectal cancer tissues and was significantly higher compared to adjacent tissues [36]. Also, other authors report that both, VASH-1 and VASH-2, expression showed a positive correlation with VEGF-A expression [37]. Correlations between these factors have been found in other types of disorders, such as diabetic retinopathy, diabetic mellitus, and cirrhosis [24, 38, 39]. This is in contrast with the results of our studies, which found no correlation in the study group. Although lack of correlation between these two factors has been observed by some authors too [40], we also found a negative correlation between them in the control group, which is different to all other published studies. One possible reason for this difference is that contrary to our studies focusing on the blood levels of these factors, most of the quoted results of other authors come from the solid tissues. Another reason is that mostly the quoted results come from ill patients and not healthy individuals. Research by Sato et al. [38] showed significantly lower concentration of VASH-1 in blood plasma than in tissue, which might explain the results obtained by us, but the study by Ren et al. [24] on the other hand found a correlation between VASH-1 and VEGF in the blood in all patient groups, ill and healthy. This indicates that some other factors might play role in the levels of these markers in the blood. Because of the limitation of our study, specified further in the last section of this article, we suspect that one of the factors might be the age. Therefore, more studies are needed to investigate this issue also because there is a lack of information on the normal range levels of these factors in the blood, which we noticed while performing literature review. When planning to use these markers as either diagnostic or prognostic factors such range must be established.

Vascular endothelial growth factor A, also known as VEGF-A, is a growth factor involved in the formation of new blood vessels and a key mediator of physiological and pathological angiogenesis. To promote neovascularization, this factor must bind to a receptor on the cell surface. There are 3 main receptor subtypes with tyrosine kinase activity to which VEGF binds: VEGFR-1 (FLT1), VEGFR-2 (FLK1/KDR), and VEGFR-3 (FLT4). The main factor initiating VEGF production is hypoxia through activation of the hypoxia inducible factor (HIF) in tumor cells. The participation of VEGF has been proven in the pathogenesis of various diseases, not only of neoplastic nature [41–43]. The pathogenesis of neoplastic disease based on the assessment of angiogenesis and the role of VEGF in this process has been proven in numerous studies and seems to be significant [42, 44, 45]. The influence of VEGF on the course of neoplastic disease and the risk of metastasis has been extensively studied, but the mechanism of initiating metastatic changes has not been fully elucidated [42]. High levels of this factor in the blood have been demonstrated in numerous cases of neoplastic diseases, such as colorectal, breast, esophageal, uterine, ovarian, bone, and prostate cancer [42]. The use of VEGF as a target for anticancer therapy that inhibits angiogenesis has become the most important aspect in oncology, the so-called antiangiogenic therapy. Bevacizumab is one of the monoclonal antibodies widely used in treatment combined with chemotherapy of various malignancies (including colon or rectal cancer, breast, lung, kidney, advanced ovarian cancer, fallopian tube, and primary peritoneal cancer). By binding to VEGF, this drug inhibits its binding to receptors (VEGFR-1 and VEGFR-2) on the surface of endothelial cells [42, 43]. It has been confirmed that high VEGF expression coexists with lower survival in patients with malignant form of inter alia, colon, rectal, and kidney cancers [42].

The process of angiogenesis in neuroendocrine neoplasms based on the assessment of VEGF concentration has been extensively studied [26, 41]. In neuroendocrine neoplasms, a strong expression of not only VEGF but also of the receptors such as VEGFR-1 and VEGFR-2 was found in relation to the surrounding tissues [42]. In the case of our work, no statistically significant differences or correlations between VEGF-A and the tested parameters were found. Similarly, Kędzierska et al. demonstrated preliminarily lack of significantly elevated concentration levels of VEGF among patients with other than cancer types of gastrointestinal malignancies, such as NETs [46]. Molecularly targeted therapy is applicable in the treatment of neuroendocrine tumors and uses the role of the VEGF-related angiogenesis pathway. There are two drugs used for this purpose: sunitinib and everolimus, which are used in the treatment of advanced pancreatic G1 and G2 NETs in the progressive phase of the disease. Sunitinib has a wide range of activity. It is, inter alia, an inhibitor of tyrosine multikinases and it is active against vascular endothelial growth factor receptors (VEGFR-1, VEGFR-2, VEGFR-3), and platelet-derived growth factor receptors alpha and beta (PDGFR-*α* and PDGFR-*β*). Everolimus has an inhibitory effect on the activity of the mTOR protein kinase, which has antiangiogenic effect [2, 46, 47].

Further studies focusing on the assessment of the mechanism of lymph node metastasis in patients with pancreatic neuroendocrine neoplasms are promising. Chang et al. studied the relationship between certain proto-oncogenic factors and the formation of metastasis through the lymphatic system in animal models. The authors suggested that c-Myc is overexpressed in the case of pancreatic NETs, which is also associated with the VEGF pathway and increased expression and secretion of vascular endothelial growth factor C (VEGF-C) [49].

This is the first study of the VASH-1 concentration analysis in the blood serum in a group of patients with neuroendocrine neoplasms. To evaluate a broader role of VASH-1 and VEGF-A in the pathogenesis of neuroendocrine tumors, we plan to expand our study, e.g., include also other factors. Our intention is the follow-up of patients and further research to verify the response to therapy and estimation of disease free and overall survival in patients with NEN.

## 5. Conclusions

Patients with neuroendocrine tumors showed lower serum level of VASH-1 in comparison to healthy volunteers. The highest level of VASH-1 was observed in tumors with localization in the pancreas. This observation might reflect the relevant function of VASH-1 in neuroendocrine tumors and deserves further evaluation to expand knowledge about angiogenesis in neuroendocrine tumors. Furthermore, the serum concentration of VEGF-A showed no statistical differences in the study group and probably does not have diagnostic importance in patients with NEN.

### 5.1. Limitations of the Study

The study showed statistically significant differences in age between the study group and the control group (*p* < 0.05). According to Hinamoto et al., the plasma concentrations of VASH-1 were inversely correlated with age [25]. According to other authors, no significant influence of age on the examined parameters for VEGF-A has been described [50–52]. While one of the limitations of this study is the significant difference in the mean age between examined groups, still we have extensively researched and proved lack of any significant associations between this parameter and concentrations of both tested factors.

## Figures and Tables

**Table 1:** Characteristics of the study and the control group with the *p* values of the Kruskal-Wallis test.

		Study group	Control group	*p* value
Number of patients, *n* (%)		120 (100%)	69 (100%)	—
Age (years), mean ± SD		57.7 ± 14.0	45.3 ± 15.2	0.001
BMI (kg/m^2^), mean ± SD		26.3 ± 5.1	24.7 ± 3.9	0.245
Gender, *n* (%)	Female	69 (57.5%)	45 (65.2%)	0.493
	Male	51 (42.5%)	24 (34.8%)	
Smoking, *n* (%)	Yes	14 (11.7%)	19 (27.5%)	0.065
	No	106 (88.3%)	50 (72.5%)	
Localization of the primary tumor for GEP-NET cases, *n* (%)	Pancreas	10 (8.3%)	—	—
	Duodenum	40 (33.3%)	—	—
	Small intestine	5 (4.2%)	—	—
	Rectum	21 (17.5%)	—	—
	Appendix	14 (11.7%)	—	—
	Stomach	4 (3.3%)	—	—
	Unknown	6 (5.0%)	—	—
Localization of the primary tumor for BP-NET cases, *n* (%)	Lung	20 (16.7%)	—	—
Histological grade for GEP-NET cases, *n* (%)	NET G1	67 (55.8%)	—	—
	NET G2	29 (24.2%)	—	—
	NET G3	4 (3.3%)	—	—
Histological type for BP-NET cases, *n* (%)	Typical carcinoid (TC)	14 (11.7%)	—	—
	Atypical carcinoid (AC)	5 (4.2%)	—	—
	Large cell neuroendocrine carcinoma (LC-NEC)	1 (0.8%)	—	—
Metastasis, *n* (%)	Total	53 (44.2%)	—	—
	Lymph nodes	42 (35.0%)	—	—
	Liver	34 (28.3%)	—	—
	Bones	7 (5.8%)	—	—
Clinical stage, *n* (%)	Stage I	46 (38.33%)	—	—
	Stage II	17 (18.33%)	—	—
	Stage III	22 (18.33%)	—	—
	Stage IV	35 (29.17%)	—	—

**Table 2:** Concentrations of the parameters analyzed in the study and the control group with the *p* values of the Kruskal-Wallis test.

	Study group	Control group	*p* value
VASH-1	219 ± 359.8 (pg/ml)	973.1 ± 1239.4 (pg/ml)	0.009
VEGF-A	326 ± 309.4 (pg/ml)	290.7 ± 175.5 (pg/ml)	0.658
Glucose	109 ± 44.2 (mg/dl)	101.8 ± 49.2 (mg/dl)	0.242
Total cholesterol	208 ± 51.0 (mg/dl)	—	—
Triglycerides	108 ± 75.0 (mg/dl)	—	—
Chromogranin A	153 ± 250.3 (*μ*g/l)	—	—
Serotonin	265 ± 366.5 (ng/ml)	—	—
5-Hydroxyindole acetic acid	7 ± 14.7 (mg/24 h)	—	—

## Data Availability

The data used to support the findings of this research are available upon request from the corresponding author, Janusz Strzelczyk: janusz.strzelczyk@sum.edu.pl.

## Supplementary Materials

Supplementary 1 Table S1: Spearman rank correlation coefficients and *p* values for the study group.

Supplementary 2 Table S2: Spearman rank correlation coefficients and *p* values for the control group.
