# Supplementary material for: Assessment of the Concentration of Endogenous Factors Regulating Angiogenesis, VASH-1 and VEGF-A, in the Blood Serum of Patients with Neuroendocrine Neoplasms
Source: Biomed Res Int. 2022 Mar 10;2022:9084393. doi: 10.1155/2022/9084393 (PMC8966743; doi:10.1155/2022/9084393)
Supplement: Supplementary 1 — Table S1: Spearman rank correlation coefficients and p values for the study group. [file 9084393.f1.pdf]

Table S1. Spearman rank correlation coefficients and p-values for the study group.

**Study group**	VASH-1		VEGF-A		Age		BMI		Chromogranin A		Serotonin		5-hydroxyindole acetic acid		Glucose		Total cholesterol		Triglycerides	
	r_S	p	r_S	p	r_S	p	r_S	p	r_S	p	r_S	p	r_S	p	r_S	p	r_S	p	r_S	p
VASH-1	1.00	0.000	-0.08	0.361	0.11	0.244	0.09	0.312	-0.02	0.847	**-0.19**	**0.036**	0.07	0.469	**0.19**	**0.038**	-0.02	0.825	0.01	0.904
VEGF-A	-0.08	0.361	1.00	0.000	-0.01	0.880	0.05	0.584	0.11	0.232	-0.04	0.636	-0.06	0.502	-0.05	0.561	0.05	0.567	0.16	0.091
Age	0.11	0.244	-0.01	0.880	1.00	0.000	**0.33**	**0.000**	0.14	0.143	0.03	0.711	0.01	0.885	**0.22**	**0.015**	0.02	0.815	0.12	0.204
BMI	0.09	0.312	0.05	0.584	**0.33**	**0.000**	1.00	0.000	-0.05	0.617	-0.12	0.214	0.02	0.796	**0.23**	**0.010**	0.14	0.131	**0.39**	**0.000**
Chromogranin A	-0.02	0.847	0.11	0.232	0.14	0.143	-0.05	0.617	1.00	0.000	**0.38**	**0.000**	**0.33**	**0.000**	-0.04	0.687	-0.08	0.383	0.06	0.510
Serotonin	**-0.19**	**0.036**	-0.04	0.636	0.03	0.711	-0.12	0.214	**0.38**	**0.000**	1.00	0.000	**0.40**	**0.000**	-0.04	0.633	-0.13	0.164	-0.12	0.189
5-hydroxyindole acetic acid	0.07	0.469	-0.06	0.502	0.01	0.885	0.02	0.796	**0.33**	**0.000**	**0.40**	**0.000**	1.00	0.000	0.00	0.980	-0.12	0.199	**-0.20**	**0.031**
Glucose	**0.19**	**0.038**	-0.05	0.561	**0.22**	**0.015**	**0.23**	**0.010**	-0.04	0.687	-0.04	0.633	0.00	0.980	1.00	0.000	0.09	0.336	**0.24**	**0.007**
Total cholesterol	-0.02	0.825	0.05	0.567	0.02	0.815	0.14	0.131	-0.08	0.383	-0.13	0.164	-0.12	0.199	0.09	0.336	1.00	0.000	**0.33**	**0.000**
Triglycerides	0.01	0.904	0.16	0.091	0.12	0.204	**0.39**	**0.000**	0.06	0.510	-0.12	0.189	**-0.20**	**0.031**	**0.24**	**0.007**	**0.33**	**0.000**	1.00	0.000
Clinical stage	0.01	0.931	0.02	0.865	0.08	0.383	**-0.21**	**0.021**	**0.22**	**0.016**	**0.31**	**0.001**	**0.31**	**0.000**	0.16	0.078	-0.17	0.057	-0.17	0.058
Histological grading	0.08	0.376	0.13	0.161	-0.12	0.202	-0.01	0.873	0.17	0.071	0.04	0.631	0.13	0.154	0.08	0.371	-0.14	0.131	0.01	0.933
Ki-67 proliferation index	-0.03	0.736	0.04	0.677	-0.06	0.491	0.06	0.521	**0.22**	**0.014**	**0.18**	**0.047**	0.11	0.214	0.07	0.418	-0.17	0.068	0.08	0.411
