# Supplementary material for: Assessment of the Concentration of Endogenous Factors Regulating Angiogenesis, VASH-1 and VEGF-A, in the Blood Serum of Patients with Neuroendocrine Neoplasms
Source: Biomed Res Int. 2022 Mar 10;2022:9084393. doi: 10.1155/2022/9084393 (PMC8966743; doi:10.1155/2022/9084393)
Supplement: Supplementary 2 — Table S2: Spearman rank correlation coefficients and p values for the control group. [file 9084393.f2.pdf]

Table S2. Spearman rank correlation coefficients and p-values for the control group.

**Control group**	VASH-1		VEGF-A		Age		BMI		Glucose	
	r_S	p	r_S	p	r_S	p	r_S	p	r_S	p
VASH-1	1.00	0.000	**-0.49**	**0.017**	0.00	0.986	-0.38	0.118	0.11	0.784
VEGF-A	**-0.49**	**0.017**	1.00	0.000	0.12	0.578	0.30	0.232	-0.50	0.172
Age	0.00	0.986	0.12	0.578	1.00	0.000	0.39	0.108	0.68	0.051
BMI	-0.38	0.118	0.30	0.232	0.39	0.108	1.00	0.000	-0.43	0.253
Glucose	0.11	0.784	-0.50	0.172	0.68	0.051	-0.43	0.253	1.00	0.000
